# The bHLH Transcription Factor *IbbHLH129* Positively Regulates the Cold Tolerance of Sweetpotato Seedlings by Modulating Auxin and Gibberellin Pathways

**DOI:** 10.3390/plants15142123

**Published:** 2026-07-09

**Authors:** Jiaquan Pan, Zitong Yang, Sitong Liu, Zhenlei Liu, Tao Yu

**Affiliations:** Tuber Division, Crop Research Institute, Liaoning Academy of Agricultural Sciences, Shenyang 110095, China; pjqamy1001@163.com (J.P.); yangzitong9870@163.com (Z.Y.); 15909824109@163.com (S.L.); l15840049311@126.com (Z.L.)

**Keywords:** sweetpotato, *IbbHLH129*, cold stress, IAA, GA

## Abstract

Low-temperature stress severely inhibits the growth and development of sweetpotato, restricts its planting geographical range, and frequently causes seedling death and yield reduction, posing a major threat to sweetpotato sustainable production. Nevertheless, the molecular regulatory mechanisms by which sweetpotato perceives and adapts to low-temperature stress have not been fully elucidated and deserve further in-depth investigation. In this study, a basic helix–loop–helix (bHLH) transcription factor *IbbHLH129* was cloned from a cold-tolerant sweetpotato variety Xs33. The IbbHLH129 protein is localized in the nucleus. *IbbHLH129* is most highly expressed in sweetpotato leaves and upregulated by low temperature, GA3, MeJA, and IAA, while repressed by ABA. Overexpressing *IbbHLH129* enhanced cold tolerance in sweetpotato seedlings under short-term cold stress through enhancing antioxidant capability and regulating hormone-related pathways. Dual-luciferase and electrophoretic mobility shift assays demonstrated that IbbHLH129 binds to the *IbYUCCA2* and *IbGID1* promoters to activate their expression. These findings suggest that *IbbHLH129* enhances cold tolerance in sweetpotato seedlings under short-term cold stress by activating IAA and GA signaling pathways. Our study provides a novel gene resource with promising applications for reducing low-temperature injury to sweetpotato seedlings cultivated in cold growing areas.

## 1. Introduction

Low-temperature stress is a critical environmental limiting factor that inhibits growth and development, and substantially reduces crop yields and adaptive distribution [1,2]. So far, a wide range of signal transduction pathways involved in cold stress response have been established. To cope with cold stress, plants have evolved a series of adaptive mechanisms, including adjustments to the membrane system, activation of the antioxidant defense system, and hormonal regulation [3]. Among these, phytohormones play a crucial role in regulating the response to cold stress. Indole-3-acetic acid (IAA) is a key plant auxin involved in regulating cold stress responses [4]. The accumulation of endogenous IAA has been shown to enhance plant tolerance to cold stress. For example, elevated endogenous IAA contents are correlated with enhanced cold tolerance in rapeseed [5]. Additionally, gibberellin (GA) has been extensively studied as a key plant hormone involved in the process used by plants to respond to cold stress. GA treatment significantly reduces the activities of antioxidant enzymes in tomato under low-temperature storage, consequently improving chilling tolerance [6]. Knocking out *WRKY53* in rice increases GA content and improves cold tolerance without a yield penalty [7]. Low temperature markedly upregulates the GA receptor *GID1*, resulting in the reprogramming of GA signaling to suppress plant growth while initiating cold stress responses [8]. Although the regulatory roles of IAA and GA in cold tolerance have been characterized in model plants and several horticultural crops, the molecular mechanisms by which IAA and GA signaling pathways jointly govern cold tolerance remain largely uncharacterized in sweetpotato.

Transcription factors (TFs) play central roles in orchestrating plant stress responses by modulating downstream gene expression [9,10]. Among these, the basic helix–loop–helix (bHLH) superfamily is the second largest TFs and has garnered attention for their versatile functions in both biotic and abiotic stress adaptation [1,11]. Accumulating evidence has highlighted the involvement of bHLHs in plant cold stress responses. For example, MdCIbHLH1 positively regulates cold tolerance in transgenic apple plants by activating *MdCBF2* expression [12]. The bHLH family member ICE1 activates transcription of the *CBF3*, while ICE1 homolog ICE2 activates transcription of the *CBF1*, positively regulating cold tolerance [13]. Overexpression of *OsbHLH002* enhances rice cold tolerance by binding to the promoter of *OsTPP1* to regulate trehalose accumulation under cold stress [14]. In Arabidopsis, overexpression of *OrbHLH001* enhances the tolerance to freezing and salt stresses [15]. Sweetpotato (*Ipomoea batatas* (L.) Lam., 2*n* = B_1_B_1_B_2_B_2_B_2_B_2_ = 6x = 90) is an economically important root and tuber crop [16]. However, its cultivation is challenged by its vulnerability to low temperatures [17]. Therefore, developing or cultivating low-temperature-tolerant sweetpotato varieties is of paramount importance for sweetpotato breeding. To date, several genes have been shown to be involved in the cold tolerance of sweetpotato [18,19,20,21,22]. However, the *IbbHLH129* gene has not been studied in sweetpotato.

In this study, we explored the functions and underlying mechanisms of *IbbHLH129* in sweetpotato. It is strongly induced by low temperatures, GA3, MeJA, and IAA, while repressed by ABA. Overexpression of *IbbHLH129* significantly enhances the cold tolerance of sweetpotato seedlings under short-term cold stress. IbbHLH129 directly activates *IbYUCCA2* and *IbGID1* by binding to the E-box in its promoter. This study offers a novel gene resource for enhancing cold tolerance in sweetpotato seedlings, which is expected to alleviate cold damage for seedling propagation in low-temperature planting regions.

## 2. Results

### 2.1. IbbHLH129 Is Involved in Cold Tolerance of Sweetpotato

**Figure 1:**
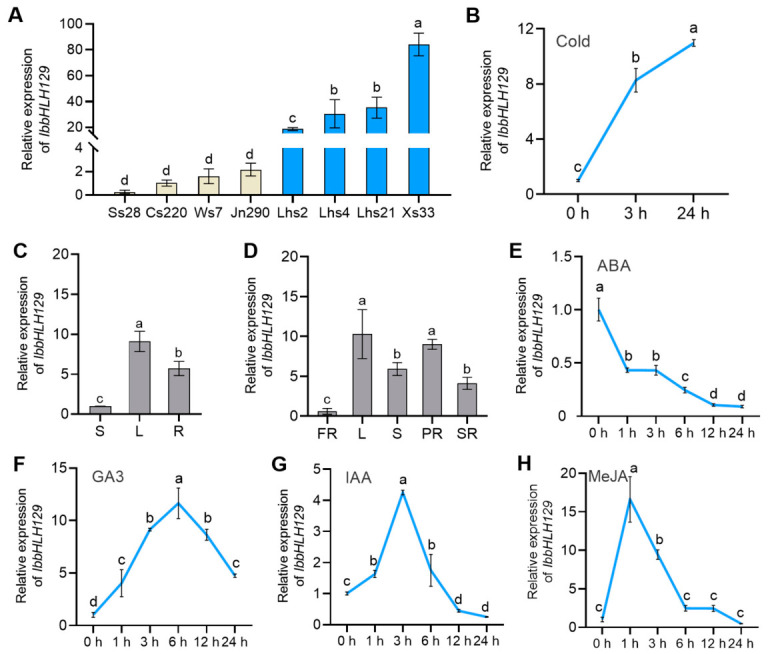
*IbbHLH129* participates in cold stress response in sweetpotato. (**A**) Expression levels of *IbbHLH129* in leaves of 30-d-old field-grown sweetpotato varieties. Ss28, Sushu28; Cs220, Chuanshu220; Ws7, Wanshu7; Jn290, Jinong290; Lhs2, Liaohanshu2; Lhs4, Liaohanshu4; Lhs21, Liaohanshu21; Xs33, Xushu33. Data are shown as mean ± SD (*n* = 3). (**B**) Expression of *IbbHLH129* in cold-tolerant variety Xs33 after different time points. Data are shown as mean ± SD (*n* = 3). (**C**) Expression of *IbbHLH129* in the stems (S), leaves (L), and roots (R) of 30-d-old in vitro-grown Xs33 plants. (**D**) Expression of *IbbHLH129* in the fibrous roots (FR), leaves (L), stems (S), pencil roots (PR), and storage roots (SR) of 120-d-old field-grown Xs33 plants. (**E**–**H**) Expression analysis of *IbbHLH129* in Xs33 plants under 100 μM ABA (**E**), 100 μM GA3 (**F**), 100 μM IAA (**G**) or 100 μM MeJA (**H**) treatments during a 24 h period. Data are shown as mean ± SD (*n* = 3). Lowercase letters indicate significant differences at *p* < 0.05 according to one-way ANOVA followed by post hoc Tukey’s test.

*IbbHLH129* (lbat.Brg.09F G019790) gene was cloned from sweetpotato cold-tolerant variety Xs33. To study the potential role of *IbbHLH129* in cold tolerance of sweetpotato, the expression level of *IbbHLH129* was analyzed. Real-time quantitative polymerase chain reaction (RT-qPCR) assay showed that the cold-tolerant sweetpotato varieties (Lhs2, Lhs4, Lhs21, and Xs33) generally exhibited higher expression of *IbbHLH129* than cold-sensitive sweetpotato varieties (Ss28, Cs220, Ws7, and Jn290) (Figure 1A). Further results showed that *IbbHLH129* was significantly induced by cold stress, with expression levels increasing by 8.27-fold at 3 h and 10.98-fold at 24 h, respectively (Figure 1B). Tissue-specific expression analysis demonstrated that *IbbHLH129* was highly expressed in the leaves of in vitro and field-grown Xs33 plants (Figure 1C,D). In addition, we characterized the hormonal responsiveness of *IbbHLH129* under abscisic acid (ABA), GA3, IAA, and MeJA treatments. The results indicated that *IbbHLH129* expression was suppressed under ABA treatment but was induced 11.64-fold (at 6 h), 4.24-fold (at 3 h), and 16.61-fold (at 1 h) under GA3, IAA, and MeJA treatments, respectively (Figure 1E–H).

### 2.2. Sequence Analysis and Subcellular Localization of IbbHLH129

**Figure 2:**
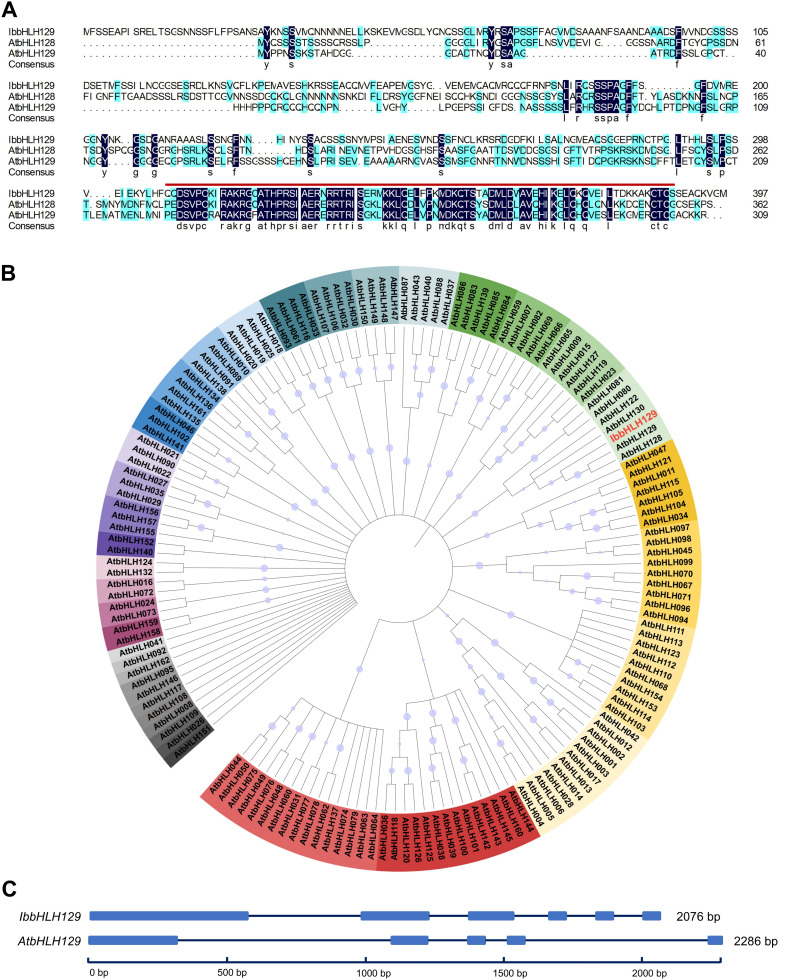
Sequence analysis of IbbHLH129. (**A**) Multiple alignments of IbbHLH129 with its homologs from other plant species with conserved amino acids shaded in different colors. The solid red line represents the conserved bHLH domain. (**B**) Phylogenetic tree of IbbHLH129 and bHLH family of Arabidopsis. The size of the circles on the nodes of the tree indicate the confidence coefficient from 1000 replicates. (**C**) Comparison of genomic structures of *IbbHLH129* and *AtbHLH129*. Boxes indicate exons and lines indicate introns.

**Figure 3:**
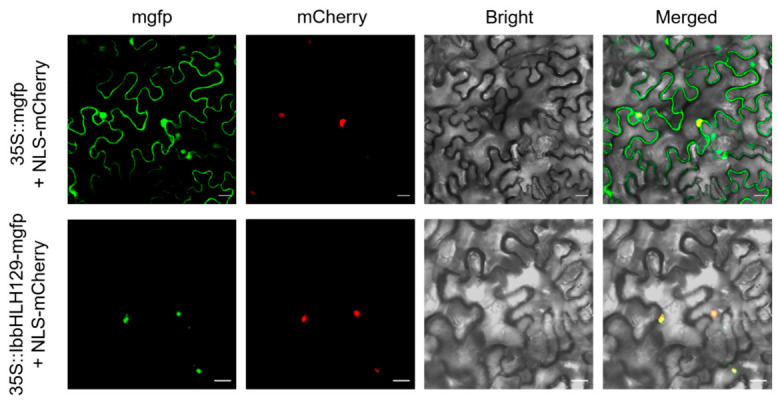
Subcellular localization of IbbHLH129. *Nicotiana benthamiana* leaves were transformed with the *IbbHLH129*-*mgfp* and the nuclear marker NLS-mCherry. Scale bars, 20 μm.

IbbHLH129 belongs to the bHLH transcription factor family, which contains one conserved bHLH domain (Figure 2A). IbbHLH129 shared the closest phylogenetic relationship with AtbHLH129 among Arabidopsis homologs (Figure 2B). The 2076 bp genomic sequence of *IbbHLH129* contained 6 exons and 5 introns, different from that of *AtbHLH129* having 5 exons and 4 introns (Figure 2C). Subcellular localization analysis revealed that the fluorescence signal of IbbHLH129 overlapped with the nuclear marker NLS-mCherry, confirming that IbbHLH129 was localized in the nucleus (Figure 3).

### 2.3. Overexpression of IbbHLH129 Enhances Cold Tolerance in Sweetpotato Seedlings

**Figure 4:**
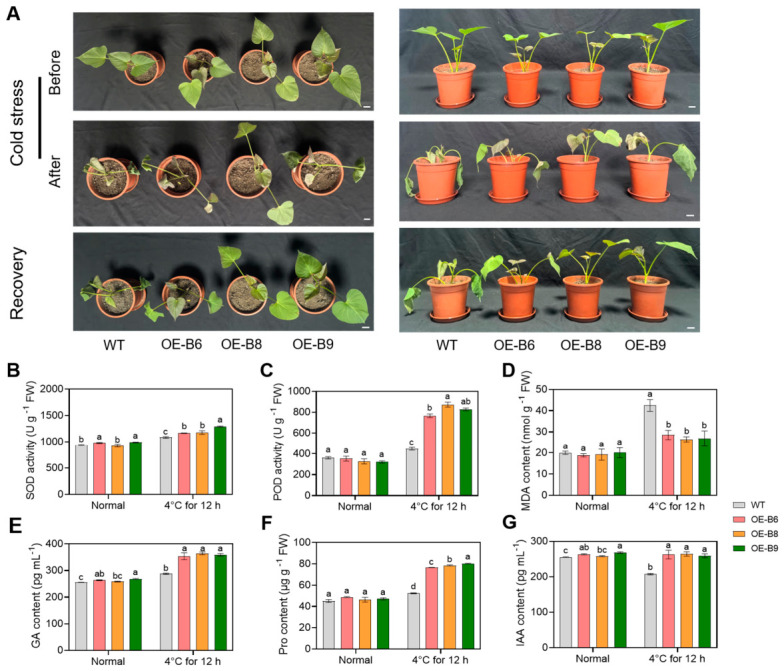
*IbbHLH129* positively promotes cold tolerance in sweetpotato (**A**) 4 °C treatment assays of the WT and *IbbHLH129*-overexpressing sweetpotato plants; “Before” represents before treatment at 4 °C, “After” represents 24 h after treatment at 4 °C, and “Recovery” represents 24 h after recovery at 25 °C. Scale bars, 2 cm. (**B**) SOD activity. (**C**) POD activity. (**D**) MDA content. (**E**) GA content. (**F**) Proline content. (**G**) IAA content. “Normal” represents a standardized climate-controlled condition at 25 °C. Data are shown as mean ± SD (*n* = 3). Lowercase letters indicate significant differences at *p* < 0.05 according to one-way ANOVA followed by post hoc Tukey’s test.

To confirm whether *IbbHLH129* contributes to cold tolerance in sweetpotato seedlings, *IbbHLH129* was introduced into sweetpotato variety Yanshu25, and 11 *IbbHLH129*-overexpressing lines (OE-B1 to OE-B11) were generated (Figure S1A–F). *IbbHLH129* exhibited a significantly increased expression level in the overexpression lines compared with the wild type (WT). Subsequently, 3 overexpression lines (OE-B6, OE-B8, and OE-B9) were selected for further studies, as these lines exhibited significantly upregulated *IbbHLH129* expression with an approximately 500-fold increase (Figure S1G). Furthermore, the overexpression and WT plants were treated at 4 °C and restored at 25 °C. The degree of wilting in the overexpression lines at 24 h under cold stress was lower compared to that of WT. Additionally, the overexpression lines recovered more rapidly than WT when returned to 25 °C (Figure 4A). To further investigate the cold tolerance pathway mediated by *IbbHLH129* in sweetpotato seedlings, the contents of stress response-related components were measured. Under 4 °C treatments for 12 h, the activities of superoxide dismutase (SOD) and peroxidase (POD), as well as the proline content, were significantly higher in *IbbHLH129*-overexpressing leaves than in the WT (Figure 4B,C,F), whereas the malondialdehyde (MDA) content was significantly lower (Figure 4D). Additionally, the levels of GA and IAA were significantly increased in *IbbHLH129*-overexpressing leaves under 4 °C treatments (Figure 4E,G). These results suggest that *IbbHLH129* positively regulates cold tolerance in sweetpotato seedlings under short-term cold stress, potentially through the ROS, GA, and IAA hormone pathways.

### 2.4. IbbHLH129 Positively Regulated the Expression of Cold Tolerance-Related Genes

**Figure 5:**
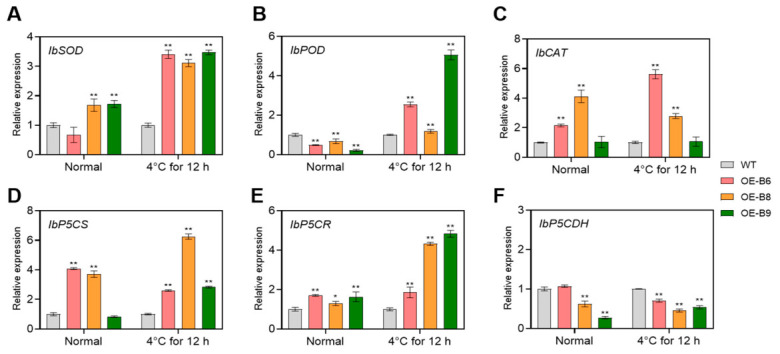
Expression analysis of cold tolerance-related genes in *IbbHLH129*-overexpressing plants. (**A**) *IbSOD*. (**B**) *IbPOD*. (**C**) *IbCAT*. (**D**) *IbP5CS*. (**E**) *IbP5CR*. (**F**) *IbP5CDH*. “Normal” represents a standardized climate-controlled condition at 25 °C. Data are shown as mean ± SD (*n* = 3). * and ** indicate a significant difference from that of WT at *p* < 0.05 and *p* < 0.01 by Student’s *t*-test, respectively.

RT-qPCR analysis was performed to detect the expression of genes related to the ROS signaling and proline biosynthesis in the *IbbHLH129*-overexpressing and WT plants. The results showed that after low-temperature treatment, the ROS signaling pathway genes *IbSOD*, *IbPOD*, and *IbCAT* were significantly upregulated in *IbbHLH129*-overexpressing plants compared with the WT (Figure 5A–C). In addition, the proline biosynthesis-related genes *IbP5CS* and *IbP5CR* were also markedly upregulated (Figure 5D,E), while the proline degradation gene *IbP5CDH* was significantly downregulated (Figure 5F).

### 2.5. Identification of IbbHLH129 Downstream Regulated Genes

**Figure 6:**
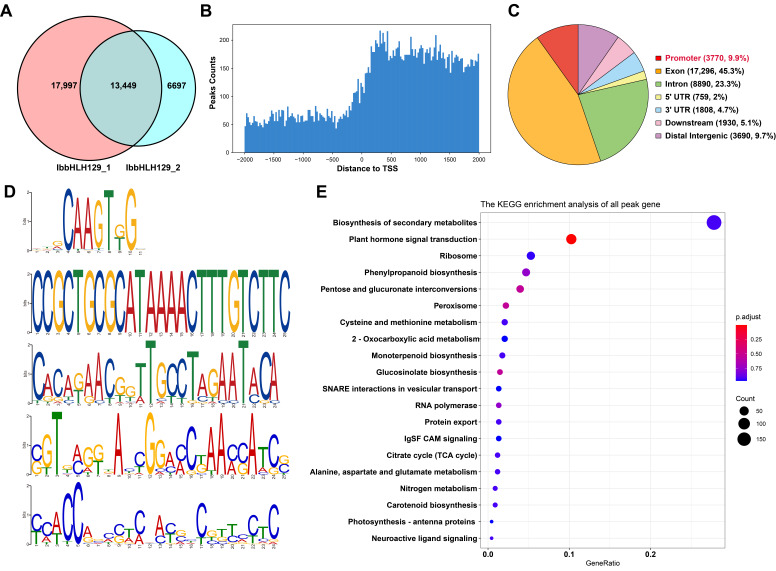
DAP-seq analysis of IbbHLH129. (**A**) Peak detection. (**B**) Peak distribution. (**C**) Peak classification. (**D**) Peak enrichment motifs. (**E**) KEGG enrichment analysis of genes associated with promoter region peaks.

The downstream target genes of IbbHLH129 were predicted via DNA affinity purification sequencing (DAP-seq) to explore the regulatory network of IbbHLH129. Sequencing results showed that a total of 13,449 peaks were identified in both replicate samples (Figure 6A). Statistical analysis of the distribution frequency of the merged peaks revealed that they were primarily distributed in the region 0–2000 bp downstream of the transcription start site (TSS) (Figure 6B). Genomic annotation of the merged peaks identified 3770 peaks mapped to promoter regions (Figure 6C). Conservative motif enrichment analysis and visualization of the peaks annotated to promoter regions revealed one highly significant and centrally enriched motif with the core sequence CAAGTG. This motif belongs to the CANNTG class of E-box binding elements, which are characteristic of the bHLH TF family (Figure 6D). KEGG enrichment analysis of the genes associated with the peaks annotated in the promoter regions revealed enrichment primarily in pathways such as biosynthesis of secondary metabolites, plant hormone signal transduction, and pentose and glucuronate interconversions, with significant enrichment in plant hormone signal transduction (Figure 6E). Of these, the biosynthesis of secondary metabolites pathway enriches a large number of core functional genes that regulate plant stress response and secondary metabolite synthesis. Collectively, these DAP-seq data demonstrate that IbbHLH129 recognizes the conserved E-box motif CAAGTG and mainly regulates a large set of genes involved in plant hormone signal transduction, laying a foundation for screening its functional downstream target genes related to cold stress response.

### 2.6. IbbHLH129 Directly Targets the Key IAA and GA Biosynthesis Genes IbYUCCA2 and IbGID1

**Figure 7:**
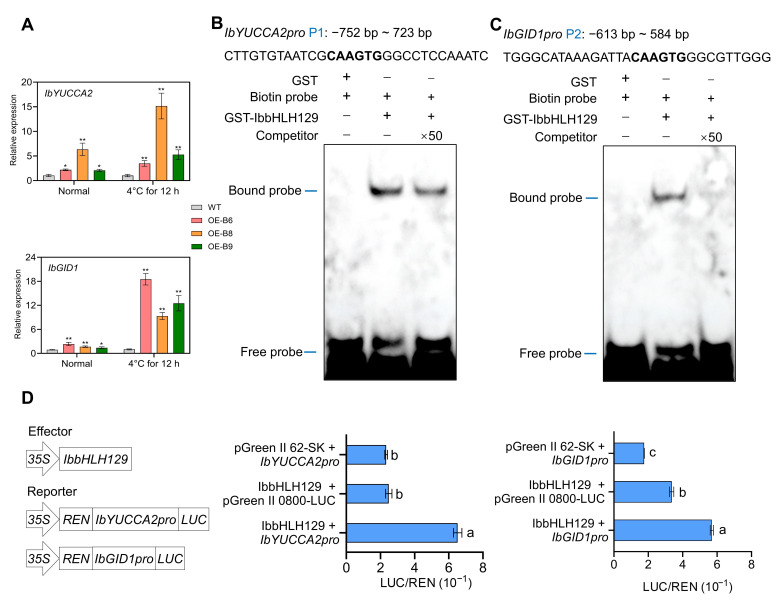
IbbHLH129 activates *IbYUCCA2* and *IbGID1* expression by directly binding to their promoters. (**A**) Expression analysis of *IbYUCCA2* and *IbGID1* in leaves of the *IbbHLH129*-overexpressing plants. Data are shown as mean ± SD (*n* = 3). * and ** indicate a significant difference from that of WT at *p* < 0.05 and *p* < 0.01 by Student’s *t*-test, respectively. (**B**,**C**) EMSA assays verified that IbbHLH129 directly targeted *IbYUCCA2* and *IbGID1* by binding to the E-box in their promoters. (**D**) Dual-LUC assay showed that IbbHLH129 activated the *IbYUCCA2* and *IbGID1* promoters. Data are shown as mean ± SD (*n* = 3). Lowercase letters indicate significant differences at *p* < 0.05 according to one-way ANOVA followed by post hoc Tukey’s test.

DAP-seq found several genes related to hormone synthesis. By combining the changes in hormone levels upon overexpression of *IbbHLH129* (Figure 4E,G), we first examined the expression of hormone biosynthetic genes in the leaves of transgenic and WT plants using RT-qPCR. The key hormone biosynthesis genes *IbYUCCA2* (encoding flavin-containing monooxygenase 2) [23] and *IbGID1* (encoding gibberellin dwarf 1) [24] were significantly upregulated in *IbbHLH129*-overexpressing plants compared with the WT (Figure 7A). Promoter sequence analysis further revealed E-box motifs in the promoter regions of *IbYUCCA2* and *IbGID1*, indicating that *IbYUCCA2* and *IbGID1* might function as downstream target genes of IbbHLH129. Subsequent EMSA and dual-LUC reporter assays confirmed that IbbHLH129 directly binds to the *IbYUCCA2* and *IbGID1* promoters and activates their expression (Figure 7B–D). Collectively, these results demonstrated that *IbbHLH129* enhances the cold tolerance of sweetpotato seedlings under short-term cold stress by activating IAA and GA signaling pathways.

## 3. Discussion

As a result of climate change, low temperature is now a primary factor restricting the yield of many crops. Sweetpotato plays a vital role in ensuring food security, yet its growth and yield are severely constrained by low-temperature injury [18,25,26,27]. Therefore, exploring the regulatory mechanisms underlying cold tolerance is essential for the genetic improvement of sweetpotato. Nevertheless, the function of bHLH family members in sweetpotato cold tolerance still needs further exploration. In this study, the novel gene *IbbHLH129* was identified in the cold-tolerant sweetpotato variety Xs33. Exogenous ABA treatment significantly repressed the expression of *IbbHLH129*, whereas cold stress, IAA, GA3 and MeJA markedly upregulated its expression (Figure 1E–H), suggesting potential antagonistic crosstalk between ABA and IAA, GA, and JA signaling pathways in regulating sweetpotato cold tolerance. Cold-induced upregulation of *IbbHLH129* presumably activates downstream cold-responsive genes to strengthen cold resistance. Given that endogenous ABA commonly accumulates under low-temperature stress [28], we hypothesize that ABA may serve as a negative feedback signal to restrict the excessive transcription of *IbbHLH129*. Such repression could avoid sustained overactivation of cold defense, reduce redundant energy expenditure and growth inhibition, and thus coordinate stress adaptation and vegetative growth through hormone homeostasis. However, since we did not characterize the expression pattern of *IbbHLH129* under combined cold and ABA treatment in the present study, the precise molecular crosstalk between cold signaling and the ABA pathway that modulates *IbbHLH129* expression still requires further verification.

Many sweetpotato planting regions in northern China and high-altitude areas frequently suffer from late spring cold and early autumn frost. Low-temperature stress often leads to seedling freezing injury, delayed seedling survival, slow vine growth and reduced field planting density, which severely restricts sweetpotato yield and restricts the expansion of suitable planting areas. Our study confirms that overexpression of *IbbHLH129* significantly enhanced cold tolerance in sweetpotato seedlings under short-term cold stress (Figure 4A). These findings highlight *IbbHLH129* as a key target for improving seedling cold tolerance in cold-sensitive sweetpotato varieties, and provide a promising candidate gene to enhance seedling cold tolerance and expand sweetpotato cultivation areas via gene-editing breeding technologies.

In plants, bHLH TFs are well established as core regulators of plant abiotic stress adaptation [29,30,31]. For instance, *NtbHLH123* enhances cold stress tolerance through direct binding to the promoters of *NtCBFs* and reactive oxidative species (ROS) scavenging-related genes to activate their expression [32]. In Arabidopsis, overexpression of either *VabHLH1* or *VvbHLH1* enhanced tolerance to cold stress [33]. Consistent with previous studies, our results demonstrated that *IbbHLH129* actively regulates the low-temperature tolerance of sweetpotato seedlings by activating IAA and GA signaling pathways (Figure 4A). bHLH transcription factors (TFs) bind to E-box cis-elements in the promoters of target genes and thereby modulate plant responses to diverse biotic and abiotic stresses [34,35]. In this study, EMSA and dual-luciferase reporter assays provided solid in vitro evidence that IbbHLH129 binds to the E-box motifs in the promoters of *IbYUCCA2* and *IbGID1* and activates their expression (Figure 7B–D). Accumulating studies have demonstrated that the endogenous plant hormones GA and IAA positively regulate plant cold stress tolerance [36,37]. Consistent with these findings, the endogenous accumulation of GA and IAA was significantly elevated in the leaves of *IbbHLH129*-overexpressing sweetpotato plants (Figure 4E,G). Although we observed obvious differences in the regrowth performance of transgenic and WT sweetpotato seedlings after cold stress and subsequent 25 °C recovery treatment, this study only recorded phenotypic variations during the recovery stage (Figure 4). Corresponding dynamic changes in *IbbHLH129* transcription and antioxidant enzyme activities were not characterized in the recovery phase. Measuring gene expression and SOD/POD activity during the recovery period would help reveal how *IbbHLH129* regulates plant repair after cold injury. Therefore, testing the molecular indicators of seedlings during the recovery stage will be an important content for our follow-up research.

The plant ROS scavenging system eliminates excess ROS by enhancing the activities of antioxidant enzymes such as SOD and POD, which protects plant cells from cold-induced oxidative damage [18,36]. For instance, PtrbHLH enhances cold tolerance by binding to E-box elements in the *POD* promoter to regulate POD-mediated H_2_O_2_ scavenging in poplar [37]. Generally, bHLH family TFs maintain cellular ROS homeostasis by modulating the expression of antioxidant enzyme genes. In the present study, the expression levels of SOD- and POD-encoding genes were markedly upregulated in *IbbHLH129*-overexpressing plants under cold treatment (Figure 5), which consequently increased SOD and POD activities (Figure 4B,C). The enhanced antioxidant enzyme activities further activated ROS scavenging pathways and alleviated cold-triggered oxidative stress, indicating that *IbbHLH129* overexpression improves cold tolerance by eliminating excessive ROS in transgenic sweetpotato seedlings. Proline confers cold resistance by mediating osmotic adjustment, stabilizing cell membranes, and protecting functional proteins in plant cells [38,39]. The leaves of *IbbHLH129*-overexpressing plants accumulated more proline compared with the WT (Figure 4F). Notably, although IbbHLH129 directly regulates GA and IAA biosynthesis via binding to their target promoters, whether this transcription factor directly modulates the expression of antioxidant defense- and proline synthesis-related genes remains to be further elucidated.

**Figure 8:**
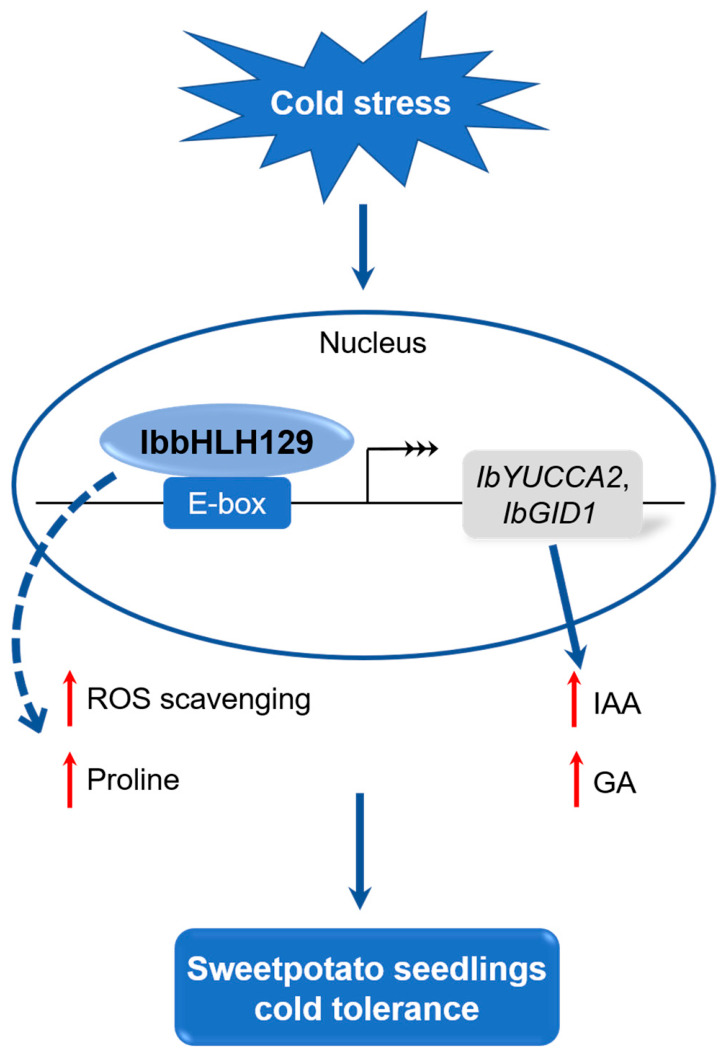
The regulatory mode of *IbbHLH129* involved in the cold stress response of sweetpotato seedlings. Arrows indicate activation. Solid lines indicate direct evidence, and dashed lines indicate putative pathways.

Collectively, these results suggest that *IbbHLH129* positively regulates cold tolerance of sweetpotato seedlings under short-term cold stress primarily by activating the GA and IAA biosynthetic pathways. The increased endogenous GA and IAA contents may further trigger downstream antioxidant defense responses to scavenge toxic ROS (Figure 8).

## 4. Materials and Methods

### 4.1. Plant Materials and Stress Treatment

The sweetpotato cold-tolerant variety Xs33 was employed to clone the *IbbHLH129* gene. The sweetpotato variety Yanshu25 was used to identify the function of *IbbHLH129*. Xs33 and Yanshu25 are preserved at the Liaoning Academy of Agricultural Sciences. These plants, cultivated in pots, were housed in a light incubator for a fortnight at 25 °C, under a light–dark cycle of 16 h light and 8 h dark, with a cool-white fluorescent light intensity of 150 μmol m^−2^s^−1^ and 75% humidity. Subsequently, these plants were relocated to a 4 °C environment for periods of 3–24 h. The third fully expanded leaf from the top was harvested, promptly immersed in liquid nitrogen, and then stored at −80 °C for future analysis. Control plants were those unexposed to the low-temperature condition. Each treatment was replicated in three separate biological instances.

### 4.2. Cloning and Sequence Analysis

Genomic DNA (Trans Gen Co., Ltd., Beijing, China) was isolated from the leaves of Xs33 according to Wang et al. [16]. Total RNA for cDNA generation was isolated from Xs33 leaves using TRIzol reagent (CoWin Biotech Co., Ltd., Beijing, China). Total RNA was reverse-transcribed into cDNA using the PrimeScript RT Reagent Kit (Takara Biomedical Technology Co., Ltd., Dalian, China). The genomic DNA elimination step was performed at 42 °C for 2 min in a 10 μL reaction containing 2 μL of 5× gDNA Eraser Buffer and 1 μL of gDNA Eraser. The reverse transcription was then carried out in a 20 μL reaction at 37 °C for 15 min and 85 °C for 5 s, using the provided RT Primer Mix (oligo dT) and PrimeScript RT Enzyme Mix. The CDS of *IbbHLH129*, excluding the stop codon, was amplified from Xs33 cDNA using specific primers and the high-fidelity MegaFit™ Pro Fidelity 2×PCR MasterMix (Applied Biological Materials Inc., Richmond, BC, Canada). The PCR reaction was performed in a 50 μL system containing 25 μL of 2× MasterMix, 2 μL each of forward and reverse primers (10 μM), 2 μL of template cDNA, and 19 μL of ddH_2_O. The amplification protocol was as follows: 94 °C for 5 min; 35 cycles of 94 °C for 30 s, 56 °C for 30 s, and 72 °C for 1 min 20 s; and followed by a final extension at 72 °C for 5 min. Gene amplification was conducted via a standard PCR instrument (LongGene Scientific Instruments Co., Ltd., Hangzhou, China).

For multiple sequence alignment analysis, the amino acid sequence of *IbbHLH129* and other bHLH homologs from Arabidopsis obtained from NCBI were aligned using the ClustalX (1.8.1) software (Conway Institute UCD, Dublin, Ireland). Phylogenetic analysis was performed with MEGA 11.0 software. An exon/intron analysis of the *IbbHLH129* gene was conducted using the GSDS 2.0 (http://gsds.gao-lab.org/). All primers in this study are shown in Supplementary Table S1.

### 4.3. Subcellular Localization

The pCAMBIA1302-*IbbHLH129*-*mgfp* plasmid was transformed into *Agrobacterium tumefaciens* strain GV3101 (pSoup-p19). Leaves from 40-day-old *N. benthamiana* seedlings were selected for infiltration. At 48 h post-transient expression, leaf tissues from the infiltrated zones were carefully excised using a scalpel, mounted on glass slides with a drop of distilled water, and covered with coverslips. The subcellular localization of the IbbHLH129-mgfp fusion protein was observed using a laser scanning confocal microscope (LSM900, Zeiss, Germany). The mgfp fluorescence was excited at 488 nm, and emission signals were collected between 500 and 540 nm. The mCherry fluorescence was excited at 559 nm, with emission detected in the range of 600–680 nm. All images were processed and assembled using ZEN software (version 3.6; Carl Zeiss Microscopy GmbH, Jena, Germany) and Adobe Illustrator (version 2026; Adobe Inc., San Jose, CA, USA).

### 4.4. Expression Analysis

Total RNA was extracted from the leaves of 30-d-old field-grown Ss28, Cs220, Ws7, Jn290, Lhs2, Lhs4, Lhs21, and Xs33 plants; from leaf, root, and stem tissues of 30-d-old in vitro-grown Xs33 plants; from storage root, pencil root, fibrous root, stem, and leaf tissues of 120-d-old field-grown Xs33 plants; and from 30-d-old in vitro-grown Xushu33 plants treated with cold (4 °C) or 100 mM ABA, GA3, IAA or MeJA for 0, 1, 3, 6, 12 and 24 h. Expression of *IbbHLH129* was quantified using RT-qPCR and SYBR Green Master Mix (Yeasen Biotechnology (Shanghai) Co., Ltd., Shanghai, China). Total RNA was extracted from the leaves of 30-d-old field-grown *IbbHLH129*-overexpressing plants treated at 4 °C for 12 h to analyze expression levels of cold tolerance-related genes with RT-qPCR. RT-qPCR was performed on a QuantStudio™ 6 Flex real-time quantitative PCR system (Thermo Fisher Scientific, Waltham, MA, USA). Each 10 μL reaction mixture contained 5 μL of 2× HQ SYBR qPCR Mix (Zoman Biotechnology Co., Ltd., Beijing, China), 0.2 μL forward primer (10 μM), 0.2 μL reverse primer (10 μM), and 1 μL cDNA template. Nuclease-free double-distilled water (ddH_2_O) was used to adjust the final reaction volume to 10 μL. The thermal cycling program was set as follows: initial denaturation at 95 °C for 30 s, followed by 40 cycles of 95 °C for 10 s and 60 °C for 35 s. The sweetpotato *IbActin* gene (AY905538) served as an internal control. All of the sweetpotato varieties used above are preserved at the Liaoning Academy of Agricultural Sciences. Three independent biological replicates were performed, each with 3 plants.

### 4.5. Production of Transgenic Sweetpotato Plants

The pCAMBIA1302-*IbbHLH129*-*mgfp* construct was transformed into *A. rhizogenes* strain K599. These clones were dispersed into 200 mL of LB broth and cultured overnight to an OD_600_ of one. Subsequently, the bacteria were centrifuged and resuspended in a buffer solution (10 mM MES and 10 mM MgCl_2_). Construct of *IbbHLH129* was introduced into the sweetpotato variety Yanshu25 via one-step *Agrobacterium*-mediated transformation as described previously [40]. The stems of field-grown sweetpotato variety Yanshu25 were cut 20 cm from the tip, and the cut seedlings were then dipped in *A. rhizogenes* for 1 h. The inoculated plants were incubated in a greenhouse for 3 d and then transplanted into the field. Storage roots were harvested, and transformed roots were identified. Transgenic sweetpotato plants were identified by PCR and RT-qPCR analyses of leaves from plants grown in vitro for 30 d.

### 4.6. Measurement of Cold Tolerance Indices

In this experiment, we set the 4 °C cold treatment for 12 h primarily based on our preliminary laboratory explorations and previous relevant study on the cold tolerance of sweetpotato seedlings [41]. Under this treatment condition, obvious phenotypic differences between WT seedlings and *IbbHLH129*-overexpressing lines were clearly observed, which facilitated an accurate comparison of physiological differences among the tested materials. Leaf tissues of WT and *IbbHLH129*-overexpressing sweetpotato plants were harvested after cold stress treatment and immediately frozen in liquid nitrogen for subsequent physiological index detection. The activities of SOD and POD, as well as the contents of proline and MDA, were quantified using corresponding commercial assay kits (Cominbio, Suzhou, China) strictly according to the manufacturer’s protocols. The GA and IAA contents in the leaves of transgenic and WT plants were determined according to the manufacturer’s instructions (Ruixinbio, Quanzhou, China). All measurements were performed with three independent biological replicates, each consisting of 6 plants.

### 4.7. DAP-Seq

A DAP-seq genomic DNA library was constructed using Xs33 seedlings as experimental material. The library was incubated with the IbbHLH129 protein under in vitro conditions to facilitate binding. DNA fragments bound to the target protein were enriched via affinity purification, and the enriched products were subjected to high-throughput sequencing using a PE150 sequencing strategy (Bluescape Scientific Co., Ltd., Shijiazhuang, China) [42,43]. Peaks from two biological replicates (*p* < 0.05, Fold enrichment > 2) were merged (MACS3), annotated with HOMER [44], and motifs identified via MEME.

### 4.8. Dual-Luciferase Assay

*IbbHLH129* was integrated into the pGreenII62-SK vector using *Pst* I and *Kpn* I sites to form the *IbbHLH129-*SK plasmids. The promoters of *IbYUCCA2* and *IbGID1* were separately inserted into the pGreenII0800-LUC vector using the same restriction sites to drive the expression of the *LUC* reporter gene. Subsequently, the above recombinant plasmids were co-transfected into rice protoplasts following a previously described protocol [45].

### 4.9. Electrophoretic Mobility Shift Assay

*IbbHLH129* was cloned into the pGEX-6P-1 vector using *Bam*H I and *Eco*R I sites to form the pGEX-6P-1-*IbbHLH129* plasmids. The recombinant plasmid pGEX-6P-1-*IbbHLH129* was transformed into *Escherichia coli* DE3 competent cells for the expression of recombinant GST-IbbHLH129 protein [46]. Biotin-labeled and unlabeled probes at the 5′ end were used as binding probes and competitive probes, respectively. The probe sequences are listed in Supplementary Table S1. EMSA was conducted using a Chemiluminescent EMSA Kit (Beyotime Biotechnology Co., Ltd., Shanghai, China) in strict accordance with the manufacturer’s instructions.

### 4.10. Statistical Analysis

Data are analyzed using one-way ANOVA followed by post hoc Tukey’s test or Student’s *t*-test at *p* < 0.05 or *p* < 0.01.

## 5. Conclusions

In summary, *IbbHLH129* is a nuclear-localized bHLH transcription factor that positively regulates cold tolerance in sweetpotato seedlings under short-term cold stress. This regulation was characterized specifically at the seedling stage. IbbHLH129 directly targets *IbYUCCA2* and *IbGID1*, thereby modulating IAA and GA biosynthesis and signaling pathways (Figure 8). This study provides mechanistic insight into the role of bHLH transcription factors in regulating short-term cold stress responses in sweetpotato seedlings. It also identifies *IbbHLH129* as a promising candidate gene for improving cold stress responses at the early developmental stage in sweetpotato breeding programs.

## Data Availability

The original contributions presented in this study are included in the article. Further inquiries can be directed to the corresponding author.

## Supplementary Materials

The following supporting information can be downloaded at: https://www.mdpi.com/article/10.3390/plants15142123/s1, Figure S1: Production of *IbbHLH129* transgenic sweetpotato plants; Table S1: Sequences of the primers and probes used in this study.
