# Supplementary material for: The bHLH Transcription Factor IbbHLH129 Positively Regulates the Cold Tolerance of Sweetpotato Seedlings by Modulating Auxin and Gibberellin Pathways
Source: Plants (Basel). 2026 Jul 9;15(14):2123. doi: 10.3390/plants15142123 (PMC13414535; doi:10.3390/plants15142123)
Supplement: Supplementary file 1 [file plants-15-02123-s001.zip › Figure S1.pdf]

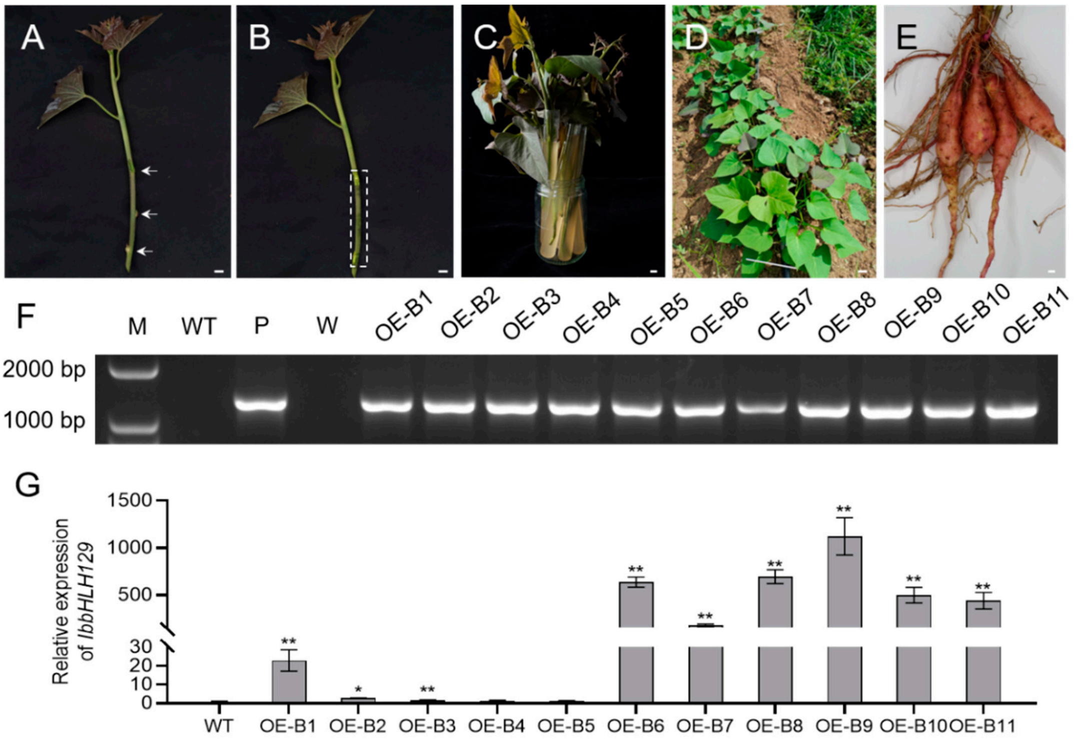

Figure S1. Production of *IbbHLH129* transgenic sweetpotato plants. (A) Stem tip cuttings (20 cm) from field-growth sweetpotato variety Yanshu25. (B) Wounding of stem before infection. (C) Wounded stem segments were immersed in *Agrobacterium* solution for infection. (D) The plants grown in a field for 30 d. (E) Sweetpotato harvested in the field. (F) PCR analysis of positive transgenic plants. Lane M: BL2000 DNA markers; Lane WT: WT as a negative control; Lane P: pCAMBIA1302-*IbbHLH129*-*mgfp* as a positive control; Lane W: water as a negative control. (G) Expression levels of *IbbHLH129* in transgenic and WT plants. Values are means ± SD (n = 3). * and ** indicate a significant difference from that of WT at $P < 0.05$ and $P < 0.01$ by Student's t-test, respectively. Scale bars, 1 cm.
