# Supplementary material for: The bHLH Transcription Factor IbbHLH129 Positively Regulates the Cold Tolerance of Sweetpotato Seedlings by Modulating Auxin and Gibberellin Pathways
Source: Plants (Basel). 2026 Jul 9;15(14):2123. doi: 10.3390/plants15142123 (PMC13414535; doi:10.3390/plants15142123)
Supplement: Supplementary file 1 [file plants-15-02123-s001.zip › Table S1.pdf]

Table S1. Sequences of the primers and probes used in this study.

Primer name	Primer sequence (5′-3′)
**Primers for genomic DNA**	
IbbHLH129-GF	ATGTTTAGCTCAGAAGCTCCGA
IbbHLH129-GR	TTATTGCATTCCAACCTTTTGAG
**Primers for RT-qPCR**	
IbbHLH129-F	CCAGAGAGTTGACCAGTGGCAG
IbbHLH129-R	CGTTGGCGGCTGAGAAATTGG
IbActin-F	AGCAGCATGAAGATTAAGGTTGTAGCACT
IbActin-R	GGAAAATTAGAAGCACTTCCTGTGAAC
IbSOD-F	TCCTGGACCTCATGGATTTC
IbSOD-R	GCCACTATGTTTCCCAGGTC
IbPOD-F	TTCACGACTGCTTCGTTGA
IbPOD-R	TTCTCAACCGCGGTCTTAA
IbCAT-F	ACGCAATTCCCGGACGTGAT
IbCAT-R	AAGCCTTCCATGTGGCGGTA
IbP5CS-F	GCCTGATGCACTTGTTCAGA
IbP5CS-R	TTGAGCAATTCAGGGACCTC
IbP5CR-F	ATAGAGGCATTGGCTGATGG
IbP5CR-R	GGTAGTCCCACCTGGTGATG
IbP5CDH-F	TCAGTTACAAGCGTTCACACAGT
IbP5CDH-R	TGCAACTTTAATAAATGACTCCCCA
IbYUCCA2-F	ATGTGTGCCGGTGGCTTATC
IbYUCCA2-R	CCACCAAAACTTTGCAGTCGT
IbGID1-F	AGGGGTTAAGTTCCCAAAG
IbGID1-R	AGAGTGCGCAAAATTCACGA
**Primers for subcellular localization and overexpression vectors**	
1302-*IbbHLH129*-F	ATGTTTAGCTCAGAAGCTCCGA
1302-*IbbHLH129*-R	ATTGCATTCCAACCTTTTGAG
**Primers for identifying overexpression plants**	
IbbHLH129-JDF	GATGTGATATCTCCACTGACGT
IbbHLH129-JDR	TTGCATACCAACCTTCTGAGC
**Primers for luciferase assay**	
pGreenII62-SK-*IbbHLH129*-F	CCGCTCTAGAACTAGTGGATCCATGTTCTCCAGCGAGGCTC
pGreenII62-SK-*IbbHLH129*-R	TGATTTCAGCGAATTGGTACCTTGCATACCAACCTTCTGAGC
pGreenII0800-LUC-*IbYUCCA2*-F	CTATAGGGCGAATTGGGTACCGTATTGCCTTCCTTAAAAAAAAGTAAT
pGreenII0800-LUC-*IbYUCCA2*-R	CGCTCTAGAACTAGTGGATCCAAAGATGCATAACTATGTCCTAGTAGT
pGreenII0800-LUC-*IbGID1*-F	CTATAGGGCGAATTGGGTACCTATTCCCACTGATATTTTGTTTTTCT

pGreenII0800-LUC-*IbGID1*-R	CGCTCTAGAACTAGTGGATCCACGGAGACAAAGTGGGAGA
**Probe for EMSAs**	
IbYUCCA2p-wt-biotin-F	CTTGTGTAATCGCAAGTGGGCCTCCAAATC
IbYUCCA2p-wt-F	CTTGTGTAATCGCAAGTGGGCCTCCAAATC
IbYUCCA2p-wt-R	GATTTGGAGGCCCACTTGCGATTACACAAG
IbGID1p-wt-biotin-F	TGGGCATAAAGATTACAAGTGGGCGTTGGG
IbGID1p-wt-F	TGGGCATAAAGATTACAAGTGGGCGTTGGG
IbGID1p-wt-R	CCCAACGCCCACTTGTAATCTTTATGCCCA
